# Neuronal Redevelopment and the Regeneration of Neuromodulatory Axons in the Adult Mammalian Central Nervous System

**DOI:** 10.3389/fncel.2022.872501

**Published:** 2022-04-22

**Authors:** Patrick Cooke, Haley Janowitz, Sarah E. Dougherty

**Affiliations:** Linden Lab, Solomon H. Snyder Department of Neuroscience, Johns Hopkins University School of Medicine, Baltimore, MD, United States

**Keywords:** axon regeneration, spinal cord injury, neuronal injury and repair, serotonin, redevelopment, glial scar, neuromodulatory neuron

## Abstract

One reason that many central nervous system injuries, including those arising from traumatic brain injury, spinal cord injury, and stroke, have limited recovery of function is that neurons within the adult mammalian CNS lack the ability to regenerate their axons following trauma. This stands in contrast to neurons of the adult mammalian peripheral nervous system (PNS). New evidence, provided by single-cell expression profiling, suggests that, following injury, both mammalian central and peripheral neurons can revert to an embryonic-like growth state which is permissive for axon regeneration. This “redevelopment” strategy could both facilitate a damage response necessary to isolate and repair the acute damage from injury and provide the intracellular machinery necessary for axon regrowth. Interestingly, serotonin neurons of the rostral group of raphe nuclei, which project their axons into the forebrain, display a robust ability to regenerate their axons unaided, counter to the widely held view that CNS axons cannot regenerate without experimental intervention after injury. Furthermore, initial evidence suggests that norepinephrine neurons within the locus coeruleus possess similar regenerative abilities. Several morphological characteristics of serotonin axon regeneration in adult mammals, observable using longitudinal *in vivo* imaging, are distinct from the known characteristics of unaided peripheral nerve regeneration, or of the regeneration seen in the spinal cord and optic nerve that occurs with experimental intervention. These results suggest that there is an alternative CNS program for axon regeneration that likely differs from that displayed by the PNS.

## Introduction

A general view in the field of neuronal regeneration is that axons in the central nervous system (CNS) do not regenerate following injury. This appears to hold true for the axons of almost all neurons within the CNS, which do indeed have a very limited capacity to regrow after injury (Huebner and Strittmatter, 2009; Canty et al., 2013; Akassoglou et al., 2017). Traumatic brain injury, spinal cord injury, and stroke can often lead to severe lifelong physical, mental, and emotional symptoms, including paralysis, depression, attention deficits, and memory loss. Axon damage accompanies the general tissue damage to the injured area, and the lack of axon regeneration within the CNS is believed to be a major underlying reason behind the persistence of symptoms following these CNS injuries. Even when the tissue is stabilized, irreparable damage to neuronal circuitry remains. Unlike CNS neurons, those with cell bodies located within the peripheral nervous system (PNS) are able to regenerate their axons and reconstruct the lost circuitry with varying degrees of success depending on the severity of damage.

Over the past 30 years, the axon regeneration field has been seeking to uncover the mechanisms underlying peripheral axon regeneration. With these results in mind, researchers then approach the CNS with two questions: (1) Why is this regeneration not occurring within the CNS and (2) If we ectopically induce these mechanisms, will we see improved regeneration? This approach has led to the theory that CNS neurons would be able to regenerate their axons if they existed within a permissive environment. However, the CNS is not an environment permissive to axon regeneration (Pasterkamp and Verhaagen, 2006; Canty et al., 2013; Sami et al., 2020). CNS injury leads to the formation of the glial scar, which includes several inhibitory molecules and secreted proteins that prevent axon regeneration (Fitch and Silver, 2008; Bradbury and Burnside, 2019). Aside from the glial scar, other factors, such as semaphorins and myelin associated glycoproteins also act as barriers to regeneration and are expressed within healthy tissue. These inhibitory factors bind receptors expressed on extending neurites and signal growth cone collapse and the cessation of axon regrowth (Schwab, 2004; Pasterkamp and Verhaagen, 2006). In this context, scientists have attempted to stimulate CNS axon regeneration in three main ways. The first avenue of treatment is to block the inhibitory signaling, either through removal of the inhibitory extracellular signal itself or blockade of its relevant receptor (Schwab, 2004; Yiu and He, 2006; Dyck and Karimi-Abdolrezaee, 2015). Second, researchers have attempted to jumpstart CNS axon regeneration by inducing the expression of pro-regenerative genes, some of which were identified within the PNS (Ma and Willis, 2015). The final approach has been to graft permissive tissues such as peripheral nerve or neuronal progenitor cells into the lesioned area in an attempt to replace the inhibitory environment with a permissive one (Richardson et al., 1980; Fischer et al., 2020). Unfortunately, each of these treatment approaches have shown only limited success, even when used in combination (Fischer et al., 2020).

Recent discoveries offer exciting new avenues of research for the field of axon regeneration within the CNS. Over the past 30 years, various grafts have been applied to the lesion site of a spinal cord injury to promote local regeneration. The most successful of these has been the neural progenitor cell (NPC) graft, which was the first treatment that succeeded in promoting some regeneration of corticospinal tract (CST) axons (Kadoya et al., 2016). Single-cell expression profiling techniques show that this regeneration is enabled through transcriptional reversion to an embryonic-like phenotype (Poplawski et al., 2020). A similar study in the PNS aligns with these results, indicating that, following injury, both PNS and CNS neurons revert to a developmental phenotype in order to promote axon regeneration (Renthal et al., 2020). While this redevelopment strategy has been hinted at in the past, these studies provide compelling evidence at a cellular resolution that inducing the reversion to a developmental-like state in adult neurons may be the key to developing new therapies to treat CNS injury.

Interestingly, serotonin and norepinephrine expressing neurons within the CNS are able to regenerate their cranial projecting axons unaided. This unprecedented phenomenon stands in contrast to the view that the CNS is an environment non-permissive to all axon regeneration. This regeneration has been widely overlooked within the field and further investigation into this regeneration promises to shed light onto many of the outstanding questions in the field, including the role of the CNS environment in inhibiting axon regeneration and potential genetic programs that could be successful in promoting axon regeneration. What is different about these neuromodulatory neurons that affords them the capacity to regenerate their axons unaided while all other neuronal subtypes are unable to do so? Are these neurons somehow able to revert more successfully to a developmental phenotype or are they employing a different pathway for regeneration than that seen within the CNS? Answering these questions and more promises to unravel many of the long outstanding mysteries within the field of CNS axon regeneration more broadly.

## Axons in the Adult Mammalian Central Nervous System Do Not Regenerate Following Injury

### Intrinsic Barriers to Central Nervous System Axon Regeneration

**FIGURE 1:**
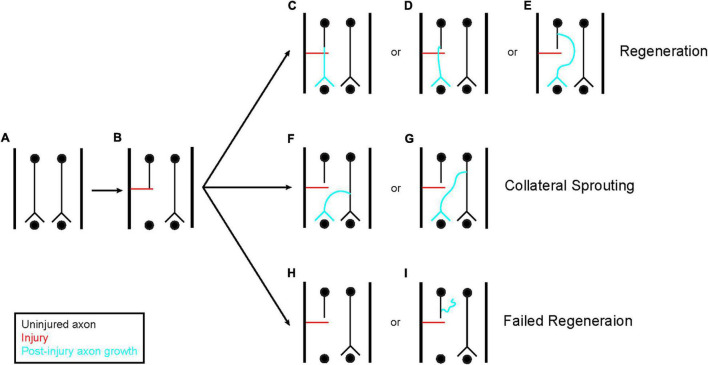
Axon regeneration and collateral sprouting. A few different classes of response can occur following axon injury **(A,B)**. First, the injured axon can regenerate **(C–E)**. This regeneration can originate directly from the transected tip **(C)** or from the axon shaft **(D,E)**. Regeneration from the axon shaft can occur close to the injured end **(D)** or from a region more remote to the injury site **(E)**. The regenerating axon does not have to pass through the injured tissue, it can also navigate around the tissue as shown in **(E)**. In addition to regeneration, axons that were spared from injury can sprout collaterals to reinnervate the denervated tissue and compensate for the damage **(F,G)**. This is a distinct process from regeneration. These collaterals can originate anywhere on the intact axon. Finally, the axon can fail to regenerate **(H,I)**. This can be observed through a complete failure to generate new growth **(H)** or new growth that fails to navigate distal to the site of injury **(I)**. These three classes of response are not mutually exclusive, each one can occur simultaneously at the same injury site across the population of injured axons.

**FIGURE 2:**
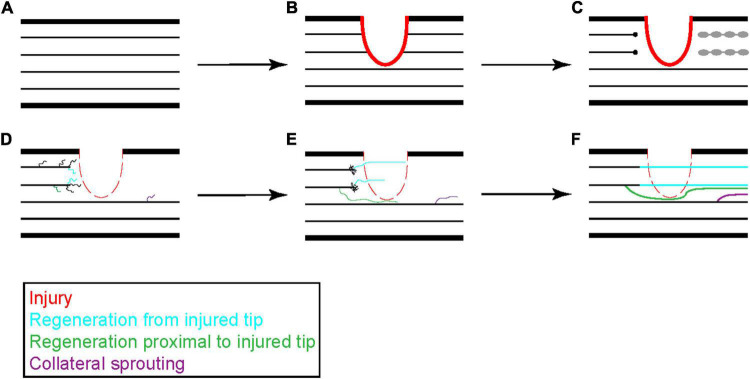
Progression of peripheral axon regeneration following injury. Following peripheral nerve injury **(A,B)**, the distal portion of an injured peripheral axon undergoes Wallerian degeneration and is cleared by invading macrophages while the proximal end retracts a short distance from the site of injury **(C)**. The proximal end of the injured axon will stabilize and form a growth cone that generates new neurites to sample the surrounding environment. These neurites can originate from the injured tip, as shown in blue, or from the axon shaft, as shown in green. Surviving axons can also undergo collateral sprouting, shown in purple, to help reinnervate distal tissues **(D)**. Neurites that receive sufficient growth factor signaling stabilize and elongate while the others retract back to the main axon shaft **(E)**. Occasionally two neurites from a single damaged axon can stabilize leading to growth from two branches. These new growths can navigate through the injured tissue or around the injured tissue to reestablish pre-injury synaptic connections **(F)**.

The disparity in regenerative potential seen by the PNS as compared to the CNS was first described by Ramon y Cajal in the early twentieth century (Cajal, 1928) and has been replicated many times since. Here, regeneration is defined as the regrowth of a severed axon, either from the severed end itself or from a new branch of the same axon generated proximal to the injured end that extends past the site of injury (Figure 1; Tuszynski and Steward, 2012). Following nerve crush injury in the PNS, the distal portion of an axon degenerates and is cleared by macrophages. The proximal end initially retracts from the lesion site back to the first Node of Ranvier and then begins a process of regeneration (Menorca et al., 2013; Sulaiman and Gordon, 2013). Initially, this regeneration consists of arborization of the axon to generate multiple outgrowing neurite branches that allow the axon to interact with the extracellular environment more broadly. Eventually a single branch will stabilize due to interactions with extracellular signaling molecules and regrow along the path left by the degenerated axon to reinnervate its target. The other neurites are then pruned (Figure 2; Cajal, 1928; Nguyen et al., 2002; Witzel et al., 2005). If the peripheral nerve is cut, rather than crushed, reinnervation of target tissue is delayed and less extensive, but still occurs in a similar manner.

The process required for regeneration is broad and multifaceted. Prior to regeneration, the neuron first undergoes a complex damage response triggered by dramatic depolarization and Ca^2+^ influx that occurs due to unregulated exposure to the extracellular environment at the lesion site. The first stage of this damage response includes resealing of the plasma membrane, often leading to the formation of a swollen dystrophic end bulb, and withdrawal from the wounded area. Following this stabilization, the axon can generate a new growth cone at its tip that will elongate and navigate to reinnervate its target (Bradke et al., 2012). Such a response requires chromatin modifications, the activation of nested transcription factor networks to induce widespread transcriptional programs, and massive cytoskeletal rearrangement reviewed in Bradke et al. (2012).

Axons within the CNS do not regenerate after injury, although some damaged axons may generate branches and extend neurites toward the lesion in a process resembling the initial stages of PNS axon regeneration. Unfortunately, this branching and outgrowth stalls before reaching the lesion site and thus does not result in regeneration following spinal cord or traumatic brain injury (Guth, 1975; Huebner and Strittmatter, 2009; Canty et al., 2013).

**FIGURE 3:**
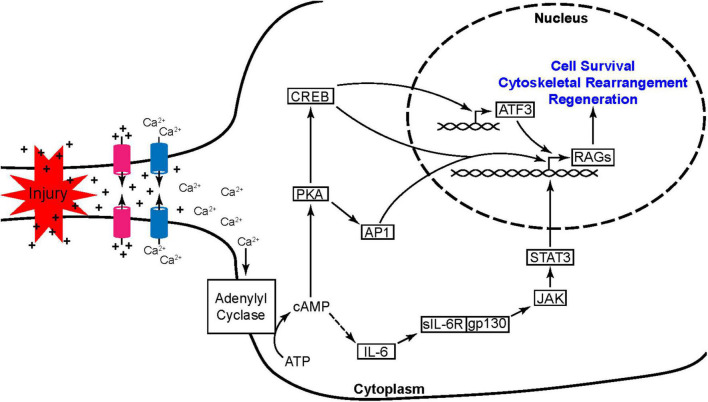
Peripheral nerve injury induces the activation of nested transcription factor networks. Axonal injury leads to the unregulated flow of ions at the breach resulting in a propagating wave of depolarization and the opening of voltage gated Ca^2+^ channels. The rise in Ca^2+^ is further potentiated by Ca^2+^ induced Ca^2+^ release from internal stores (not shown). Ca^2+^ then activates adenylyl cyclase leading to a dramatic increase in cAMP production. This rise in cAMP promotes axon regeneration through two pathways, one initiated by PKA and the other through activation of IL-6. PKA leads to the activation of transcription factor hubs AP1 and CREB while the IL-6 pathway leads to the activation of STAT3. These three transcription factor hubs induce the expression of other regeneration associated genes (RAGs) including ATF3, another transcription factor hub crucial to the early injury and regeneration response.

PNS neurons undergo dramatic transcriptional changes which are different from those seen in CNS neurons following injury. Notably, injury-evoked activation of adenylyl cyclase together with Ca^2+^ influx leads to the elevation of cyclic adenosine monophosphate (cAMP). Among other targets, cAMP activates protein kinase A (PKA), a positive regulator of two transcription factors: cAMP response element binding protein (CREB) and Activator Protein 1 (AP1). Within the vast array of genes whose transcription is altered in this response are regeneration associated genes (RAGs) (Figure 3). RAGs are genes that contribute to PNS axon regeneration, many of which can also promote partial CNS axon regrowth (Neumann et al., 2002; Qiu et al., 2002; Huebner and Strittmatter, 2009; Knott et al., 2014; Ma and Willis, 2015).

Over the years, a number of genes have been identified as contributors to PNS axon regeneration. These experiments used mutant mice or RNAi loss-of-function (LOF) strategies to knockdown expression of specific proteins in order to assess their involvement in axon regenerations. Many of these genes have subsequently been experimentally expressed within CNS neurons in an attempt to promote axon regeneration. The most successful genetic manipulations aimed at improving CNS axon regeneration are the overexpression of transcription factors that are able to modulate the expression of yet other transcription factors involved in regenerative programs, termed “hubs.” These hubs include ATF3, AP1 (c-Jun and c-Fos), C/EBPβ, KLF family members, CREB, SMAD1, SOX11, STAT3, and several others (Jenkins and Hunt, 1991; Schwaiger et al., 2000; Tsujino et al., 2000; Gao et al., 2004; Nadeau et al., 2005; Okuyama et al., 2007; Moore et al., 2009; Zou et al., 2009; Knott et al., 2014; Ma and Willis, 2015).

Inducing ectopic expression, primarily through the use of AAV transfection, of any individual hub gene in CST neurons *in vivo* does not yield impressive results for central axon regeneration following spinal cord injury- they increase initial axon arborization and can promote some process extension that is not able to cross the lesion site (Ma and Willis, 2015). Induction of multiple hubs concurrently has been shown to improve neurite outgrowth from the injured axon and increase the distance these neurites are able to regrow. One of the most successful molecular manipulations shown to improve regeneration of CNS axons *in vivo* is the injection of cAMP into neurons following injury. Injection of cAMP, or its synthetic analog, dibutyryl cAMP, into the cell bodies of injured neurons has been shown to improve axon regeneration across the lesion site following multiple injury models in several different neuronal subtypes within the CNS (Hannila and Filbin, 2008). cAMP elevation leads to the activation of CREB and AP-1, both important transcription factor hubs that promote regenerative programs (Knott et al., 2014; Ma et al., 2014). Furthermore, the results from these studies support the hypothesis that CNS neurons would be able to functionally regenerate their axons following injury if they were able to activate regenerative gene programs, as seen in the PNS.

Another strategy for promoting axon regeneration is to induce the activity of downstream pathways that directly affect axon growth. The most promising attempt has involved inhibiting phosphatase and tensin homolog (PTEN), a phosphatase which serves as a negative regulator of the mammalian target of rapamycin (mTOR) pathway. Deleting or inhibiting PTEN removes the brakes from the mTOR pathway which is involved in cell growth and proliferation and, during neuronal development, in axon extension (Ménager et al., 2004; Zhou et al., 2004; Cosker and Eickholt, 2007; Nieuwenhuis and Eva, 2022). PTEN deletion promotes regeneration through the lesion site of optic nerve and spinal cord injuries, albeit in an age-dependent manner. PTEN knock-out mice injured at 6 weeks old show impressive regeneration following both optic nerve and spinal cord injury. However, this regeneration is reduced if the mice are instead injured at 8 weeks old and further reduced if injured between 10 and 14 weeks of age (Park et al., 2008; Liu et al., 2010; Du et al., 2015; Geoffroy et al., 2016; Leibinger et al., 2016; Nieuwenhuis and Eva, 2022). Much like the studies which promoted axon regeneration through the activation of transcription factor hubs, PTEN deletion did not improve functional recovery following spinal cord injury in adult mice following T8 dorsal hemisection injury as assessed by monitoring hindlimb function Geoffroy et al., 2015; reviewed in Nieuwenhuis and Eva (2022).

While limited, inducing the expression of transcription factor hubs or the activity of downstream pathways successfully promotes partial axon regeneration in the adult mammalian CNS. This partial success indicates that these neurons harbor the required programs for axon regeneration, however, these programs are failing for some reason. These results suggest the following questions: what is preventing CNS axons from activating regenerative programs? When induced experimentally, why do these programs show some initial success before failing, is something actively inhibiting them? Do epigenetic changes following neuronal development permanently lock these genes in heterochromatin or do extrinsic signals suppress regenerative gene expression programs in CNS neurons?

### Extrinsic Barriers to Central Nervous System Axon Regeneration and Molecular Components of the Glial Scar

Following CNS injury, microglia, oligodendrocyte precursor cells, meningeal cells, and astrocytes are recruited to the injury site to form a glial scar. Astrocytes activate and secrete tenascin, semaphorin3, ephrin-B2, and chondroitin sulfate proteoglycans (CSPGs) to isolate the injury in order localize the damage and inflammation as well as to facilitate healing (Fitch and Silver, 2008; Bradbury and Burnside, 2019). Conditional deletion of STAT3 or SOCS3 within the astrocytes of adult mice, to prevent their activation, is associated with increased tissue disruption, demyelination, cell death, and a failure to repair the blood brain barrier (Okada et al., 2006; Herrmann et al., 2008; Dyck and Karimi-Abdolrezaee, 2015). Though scar formation is crucial for the acute damage response, it also may create a barrier through which axons are unable to regrow.

**FIGURE 4:**
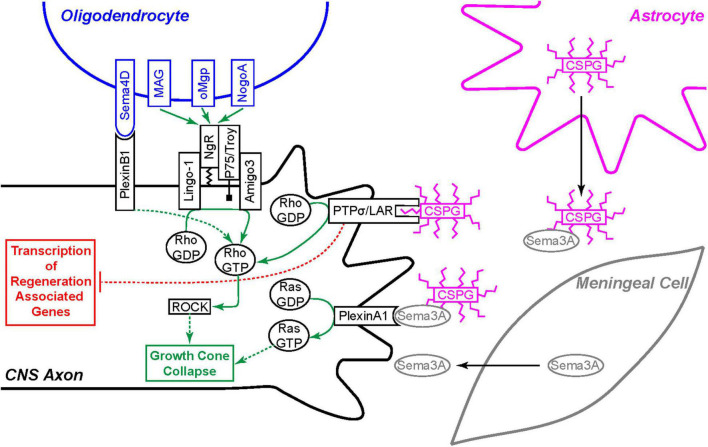
Extrinsic inhibition of CNS axon regeneration. Oligodendrocytes express four membrane bound regeneration inhibitors: Sema4D, MAG, oMgp, and NogoA. Sema4D binds to the PlexinB1 receptor and the others bind to the Nogo-66 Receptor (NgR) complex expressed on the CNS axon. Each of these receptors signal growth cone collapse through the Rho/ROCK pathway. Invading meningeal cells in the glial scar secrete Sema3A molecules which associate with chondroitin sulfate proteoglycan (CSGP) glycosaminoglycan (GAG) side-chains and bind the PlexinA1 receptor to signal growth cone collapse through the activation of Ras. CSPG GAG chains, secreted by astrocytes and other cells, bind the PTPσ or the LAR receptor to signal growth cone collapse through the same Rho/ROCK pathway. Furthermore, the PTPσ and LAR receptors also signal back to the cell body to inhibit pro-regeneration signaling cascades.

The formation of the glial scar is believed to be a major factor preventing axon regeneration within the CNS. While each of the astrocyte secreted factors previously mentioned are associated with extrinsic inhibition of regeneration, CSPGs have been the dominant focus within the field. CSPGs are a family of proteoglycans that constitute part of the extracellular matrix of the glial scar and include aggrecan, brevican, neurocan, versican, phosphocan, and NG2 (Silver and Miller, 2004; Yiu and He, 2006). In the developing nervous system, CSPGs help mark glial boundaries such as the spinal cord roof plate, optic tectum, and dorsal root entry zone to prevent ectopic growth of axons in the adult nervous system (Kubota et al., 1999; Dyck and Karimi-Abdolrezaee, 2015). CSPGs may inhibit axon regeneration by various mechanisms. The core protein of NG2 has been shown to act as an inhibitory signaling molecule, preventing axon regrowth (Ughrin et al., 2003). Additionally, the glycosaminoglycan (GAG) side chains of CSPGs have been heavily implicated in this inhibitory process. GAG side chain binding to receptor protein tyrosine phosphatase σ (PTPσ) or its subfamily member leukocyte common antigen related phosphatase (LAR) inhibits growth cone extension by activating the Rho/ROCK pathway at the growth cone and inhibiting Erk1/2 signaling in the cell body (Figure 4; Shen et al., 2009; Fisher et al., 2011; Sami et al., 2020). Additionally, GAG side chains create a dense network that can block growth by sterically hindering pro-growth adhesion molecules such as laminins and integrins, thus depriving the growth cone of the signals required for extension (Sami et al., 2020).

Treatment with Chondroitinase ABC degrades CSPG GAG chains by enzymatic digestion into smaller disaccharides, removing their growth inhibitory capability. Within the glial scar, chondrointinase treatment has been one of the most promising strategies for improving axonal regeneration following injury to both the spinal cord and optic nerve in rodents and in non-human primate models of spinal cord injury (Lemons et al., 1999; Bradbury et al., 2002; Yiu and He, 2006; Dyck and Karimi-Abdolrezaee, 2015; Rosenzweig et al., 2019). While chondroitinase ABC treatment is able to improve regeneration and some functional recovery following injury, complete recovery remains elusive. The studies mentioned here present a simplified role for CSPGs in axon regeneration. The true role of CSPGs is much more nuanced, the details of which are beyond the scope of this review (reviewed in Dyck and Karimi-Abdolrezaee, 2015; Sami et al., 2020). In short, CSPG GAG chains have pro-regeneration factors associated with them as well as those involved with inhibition. Furthermore, there appear to be differences in the molecules that associate with CSPG GAG chains based upon the cell type that secreted them. These factors likely explain why chondroitinase ABC treatment has not been able to promote axon regeneration as effectively as many had predicted.

Recent evidence suggests that the reactive astrocytic component of the glial scar may not always inhibit regeneration, as previously believed. For example, researchers can experimentally promote regeneration of a DRG sensory neuron’s central ascending branch by first applying a “conditioning lesion” to its peripheral branch. Combining a conditioning lesion with locally applied brain-derived neurotrophic factor (BDNF) and neurotrophin-3 (NT-3) stimulates regeneration of ascending sensory axons through the glial scar without any direct manipulations to degrade the scar itself (Anderson et al., 2016). When astrocyte activation was blocked in mice, using conditional knock-out of the STAT3 gene, spinal cord axons did not display increased branching or regrowth following severe spinal crush injury. On the contrary, both descending CST and ascending sensory tract axons retracted further from the lesion site than those of WT control mice. According to the glial scar hypothesis, preventing astrocyte activation should increase axon arborization and regeneration, a result not seen in this set of experiments. Interestingly, in these STAT3 conditional knock-out mice, CSPG levels remained elevated, indicating that other cells involved in the injury response may be responsible for the bulk of CSPG production. When this group examined gene expression profiles before and after spinal cord injury in STAT3 conditional knock-out mice compared to WT, they found a wide range of injury-induced changes to WT astrocytes, some known to be beneficial to regrowth. Astrocytes from injured animals harboring the conditional STAT3 knock-out remained transcriptionally similar to uninjured WT astrocytes (Anderson et al., 2016). While this set of experiments casts doubt onto the role that activated astrocytes play in obstructing regeneration, further experiments are required to address the roles of the other scar components before dismissing the glial scar hypothesis. In these experiments, CSPG levels remained elevated at the lesion site independent of astrocyte activation. Furthermore, the success that other groups have achieved in promoting neurite outgrowth following spinal cord injury using chondroitinase ABC, indicates that scar formation and the CSPGs inherent to it are likely a major barrier to regeneration. Rather it seems that astrocyte activation is not as crucial to the inhibition of axon regeneration as previously believed, and it could actually be beneficial for promoting healing and regrowth, at least in some circumstances.

Supporting the idea that the injury induced glial scar is an important barrier to regeneration, Silver and colleagues used a model of minimal injury at the dorsal root entry zone to sever the central branch of sensory axons without inducing a glial scar. These axons showed impressive regeneration into the spinal cord following injury (Davies et al., 1997). A follow up study found that the donor sensory axons grew when transplanted rostral to the lesion site of a spinal cord injury, indicating that the glial scar is indeed a major barrier to axon growth (Davies et al., 1999).

The glial scar is not the only extrinsic barrier an injured neuron faces. Canty et al. (2013) used a laser to cause microlesions that sever either a single or a small number of axons without inducing an inflammatory response or glial scar. Interestingly, following these laser-induced axotomies in the adult mouse cerebral cortex, neurons showed very limited regrowth even without having to overcome the obstacle of a glial scar. These results indicate that a non-permissive environment exists within healthy CNS tissue (Canty et al., 2013). One possible caveat is that the damage caused by laser-induced axotomy, which is not extensive enough to activate glial scarring, does not damage tissue enough to extensively activate regenerative gene programs necessary for regrowth. Additionally, a laser microlesion may create a unique barrier that has yet to be characterized.

As demonstrated by Canty and colleagues, the environment of the adult mammalian CNS is not permissive to axon regeneration even in the absence of glial scarring. One contributor to this non-permissive environment is CNS myelin. The oligodendrocytes that form myelin sheaths within the CNS express myelin-associated proteins that directly inhibit axon regeneration. The most well-known of these inhibitors are Nogo-A, myelin-associated glycoprotein (MAG), and oligodendrocyte-myelin glycoprotein (OMgp). These three myelin-associated axon regeneration inhibitors all bind with high affinity to the Nogo-66 receptor (NgR), triggering an intracellular signaling cascade that leads to the activation of RhoA, eventual stabilization of actin filaments within the growth cone, and thereby halting further elongation (Fournier et al., 2001; Domeniconi et al., 2002; Liu et al., 2002; Wang et al., 2002; Schwab, 2004). Blocking the inhibitory action of the Nogo-66 receptor is associated with improved recovery following CNS injury in both rodent and non-human primate models (Simonen et al., 2003; Schwab, 2004; Wang et al., 2020). CSPG inhibitory effects on axon regeneration converge on this same pathway through activation of RhoA (Monnier et al., 2003; Schwab, 2010; Sami et al., 2020). Unlike CSPGs from astrocytes, myelin-associated inhibitors are constitutively expressed within oligodendrocytes in the absence of injury (Pasterkamp and Verhaagen, 2006). While myelin-associated inhibitors and Nogo-66 receptor expression remains constant, molecules involved in the signaling cascade downstream of the Nogo-66 receptor are differentially regulated following CNS injury making neurons more sensitive to myelin-associated inhibition. Examples of such downstream proteins include AMIGO-3 and LINGO-1, both integral components of the Nogo-66 receptor signaling complex (Figure 4; Mi et al., 2004; Ahmed et al., 2013).

In addition to myelin-associated inhibitors, certain semaphorins (Semas) also contribute to the general inhibitory environment within the CNS. Semaphorins comprise a large gene family of both secreted and membrane-associated proteins that act as guidance cues within the developing CNS. All neurons and some glial cells within the CNS express semaphorins during development. In the adult mouse, semaphorin expression becomes restricted to a specific population of neurons. The membrane bound Sema4D is expressed on oligodendrocytes and plays a similar role to myelin associated inhibitors, contributing to the general non-permissive environment of the CNS (Pasterkamp and Verhaagen, 2006). Sema4D binding to its receptor, plexinB1, initiates a signaling pathway that leads to cytoskeletal changes and growth cone collapse through the Rho/ROCK pathway, similar to the Nogo-66 receptor (Oinuma et al., 2004). The most studied semaphorins affecting axon regeneration in the adult CNS are the secreted Sema3s (Pasterkamp and Verhaagen, 2006; Mecollari et al., 2014). In contrast to Sema4D, the Sema3s are specifically upregulated following CNS injury (Pasterkamp et al., 1999). Sema3A is the best studied of the Sema3s and colocalizes with fibrotic expression indicating that meningeal cells invading the neuronal scar are their main source. Neurons themselves may also contribute to Sema3A levels as they are known to secrete Sema3A under hypoxic conditions following ischemic events (Joyal et al., 2011). Once secreted, Sema3A molecules associate with CSPGs and signal through a holoreceptor complex made up of neuropilin and plexinA1 subunits. Like Sema4D, Sema3A binding to its neuropilin/plexin holoreceptor induces growth cone collapse, directly preventing axon regeneration through the scar (Figure 4; Pasterkamp and Verhaagen, 2006; Mecollari et al., 2014; Sami et al., 2020).

Finally, the CNS inhibitory environment is inflammation. The microglia and astrocytes recruited to the site of injury not only contribute to glial scar formation, but they also secrete cytokines which recruit non-resident immune cells like macrophages (Gaudet and Fonken, 2018). The result of this inflammatory response is complex, with evidence suggesting inflammation creates a barrier for axon regeneration (Gaudet et al., 2016; Gaudet and Fonken, 2018) but also that inflammation is sometimes necessary for axon regrowth (Kigerl et al., 2009; Harrison et al., 2017; Wang et al., 2019). The role of inflammation in regeneration is an ever-broadening field which has been extensively review elsewhere (Fitch and Silver, 2008; Benowitz and Popovich, 2011; Orr and Gensel, 2018; Estera et al., 2021; Kwiecien, 2022).

## Graft Transplants Promote Axon Regeneration Following Spinal Cord Injury

Neurite outgrowth and regeneration following spinal cord injury can be promoted through the application of various tissue grafts to the site of injury. The first studies of this type, by Aguayo and colleagues in the 1970s, used peripheral nerve and Schwann cell grafts. In a series of experiments, the group cut out tissue around the optic nerve and grafted it in multiple places throughout the PNS. Upon later examination, they found regenerated PNS axons distal to the injury site. However, the axons did not regrow through the graft but rather around it. In contrast, they found that if a PNS graft was placed in the lesion site following spinal cord injury, axons from various brainstem nuclei were able to regrow into the PNS graft, although none were seen to extend beyond the grafted tissue (Aguayo et al., 1978; Richardson et al., 1980, 1984; David and Aguayo, 1981). Somehow, the PNS environment was able to partially stimulate a regenerative state in CNS neurons whose axons were injured, despite the general inhibitory environment comprising the distance between the graft and neuronal cell bodies. These studies helped to establish the hypothesis that adult mammalian CNS neurons are able to regenerate their axons following injury if they can enter the correct regenerative state. Such a state can be achieved within the right environment, as exists in the PNS.

By the late 1980s, other groups had identified Schwann cells as a key component in the PNS graft’s ability to promote regeneration of CNS neurons following spinal cord injury (Kromer and Cornbrooks, 1985; Guénard et al., 1992). While PNS grafts were able to stimulate axon regeneration into the graft itself, axons were unable to reinnervate the region distal to the graft to any significant extent–significant reinnervation and reformation of synapses in target tissues was never seen with PNS grafts alone. Furthermore, these studies never showed axon regrowth from neurons with somata residing in the brain. Rather, the regenerating axons were all from propriospinal neurons within the spinal cord. In 1995, Xu and colleagues hypothesized that Schwann cell expression of BDNF and NT-3 was partially responsible for this regeneration. When they treated with both BDNF and NT-3 in addition to a Schwann cell graft, they not only showed a significant increase in the number of fibers that were able to extend into the grafted tissue but also identified serotonergic fibers from the raphe nuclei extending past the lesion site, marking the first observation of axon regeneration in the spinal cord from a CNS neuron situated within the brain (Xu et al., 1995). Later studies replaced Schwann cells with cultured fibroblasts genetically modified to express BDNF, suggesting that the growth factors, rather than the Schwann cells themselves, were stimulating axon regeneration (Liu et al., 1999). This approach was then further improved by combining bone marrow stromal cell grafts and the injection of growth factor expressing Lentivirus into target regions for axon regrowth. Initially, it was hoped that these bone marrow stromal cells would develop into new neurons to repopulate the injured area. While these cells did not transition into a neuronal fate, this graft helped promote tissue healing through the expression of growth factors and cytokines to support revascularization of the injured region (Parr et al., 2007). The important aspect of this method is the injection of growth factor expressing Lentivirus into the denervated tissue. The growth factors produced by injected Lentivirus diffuse, creating a concentration gradient originating from the target tissue and thus providing an instructional environment to guide the new growth cones. This approach facilitated increased axon regeneration, partial reinnervation of target tissues, and some reformation of synapses following spinal cord injury (Alto et al., 2009; Lee et al., 2013). Other experiments have paired the application of a graft with blockade of CSPG signaling (Bradbury et al., 2002; Vavrek et al., 2007; Shen et al., 2009; Lee et al., 2013). The benefits provided by these growth factors show that a permissive environment lacking extrinsic inhibitors might not be sufficient for axon regrowth. Instead, the environment may have to provide signals to promote regeneration. Growth factors gradients originating from denervated tissue could provide guidance to promote productive axon regrowth beyond that seen following interventions that only address inhibitory factors such as Chontroitinase ABC treatment or blockade of the Nogo-66 receptor intended to merely remove non-permissive signals from the environment.

These studies, and others, support the hypothesis that CNS neurons maintain the capacity to regenerate when provided with not only permissive substrates but also an environment that promotes a growth state. By adding chondroitinase to cleave CSPGs within the path of regrowing axons, inhibitory signals are removed from the environment. Adding growth factors then provides the positive, perhaps at times instructive, signals for regrowth. This favorable environment is completed by applying a bone marrow stromal cell graft or a Schwann cell graft to the lesion site providing tissue the axon can regrow through. However, a few caveats must be mentioned. First, while these methods are able to promote axon regeneration and partial behavioral recovery, full behavioral recovery as assessed by various motor related tasks following a complete spinal cord transection was not shown. These studies examined either partial transection or crush (contusion) injuries (Cheriyan et al., 2014). Furthermore, the regenerating axons do not reach target tissues and reform synapses at densities seen prior to injury. Finally, axon regeneration occurred to varying extents depending on neuronal subtype. Neurons that displayed some increased sprouting and regrowth were located within the spinal cord, reticular nucleus, raphe nucleus, locus coeruleus, and other brain stem nuclei (Jin et al., 2002; Vavrek et al., 2007; Lu et al., 2012; Lee et al., 2013). No study was able to provide definitive evidence of functional CST axon regeneration, regardless of intervention strategy. Zhigang He’s group found that CST axons extended caudal to a spinal cord hemisection, in which half the spinal cord is transected, or complete crush injury following mTOR stimulation through PTEN deletion or the enforced overexpression of insulin-like growth factor 1 in CST neurons. This regrowth was accompanied by some synaptic reformation, as observed by BDA/vGlut1 immunostaining and, following dorsal hemisection, minor improvement in hindlimb function (Liu et al., 2010, 2017; Zukor et al., 2013). While these results are promising, the incomplete nature of the hemisection injury leaves open the possibility that axon reappearance occurred due to collateral sprouting and the improvement in hindlimb function could be a result of reinforcement of surviving circuits due to repeated behavioral testing as was noted by the same group in a later study (Chen et al., 2018).

The findings discussed thus far indicate that there is more to CNS axon regrowth than just the presence of inhibitory factors and the lack of a pro-growth environment preventing the activation of the regenerative program in CNS neurons. One possibility is that these studies had not identified the correct milieu required to promote effective regrowth. However, the difference in regenerative capacity seen across neuronal subtypes indicates that there are neuron-specific considerations as well. Similar cell-type specific differences were observed examining the regeneration of retinal ganglion cell subtypes following optic nerve crush in PTEN knock down mice. PTEN knock-down to stimulate mTOR activity has been shown to be one of the most effective methods at promoting axon regeneration of RGCs, however, this regeneration seems to be dominated by α and M1 RGCs with very limited success in other subtypes (Duan et al., 2015). The disparity seen between different cell types indicates that these populations may have different requirements for activating this regenerative gene program or perhaps different regeneration programs entirely.

While the studies discussed thus far successfully demonstrated regeneration of CNS neurons, no experiment had shown direct evidence of CST axon regeneration. Previous studies had recorded motor recovery following incomplete spinal cord injuries, in which only half the spine is damaged, using behavioral tests or extracellular recordings in the spinal cord following stimulation in the motor cortex (Bregman et al., 1995; Bradbury et al., 2002). Bregman and colleagues went as far as removing the sensorimotor cortex to indicate that behavioral recovery was reliant upon regeneration of CST axons. Each of these methods indicates that these CST axons may be regenerating; however, they do not rule out the possibility that the behavioral recovery is a result of collateral sprouting from surviving axons on the undamaged side. One method to definitively show axon regeneration would be to perform a complete spinal cord transection.

The first study that showed CST axon regeneration following complete transection of the spinal cord was performed using a combined treatment of cAMP injections to the neuronal cell bodies, a bone marrow stromal cell graft to the lesion site, and the careful construction of a BDNF gradient distal to the lesion (Lu et al., 2012). What set this study apart from others was the careful construction of a BDNF gradient distal to the lesion site. Here, the authors showed that using growth factor guidance cues to create a spatially instructive environment in tandem with the permissive graft is much more effective at promoting regeneration than the graft-facilitated permissive environment alone.

To date, the most successful intervention in promoting recovery following a spinal cord injury is the application of a multipotent neural progenitor cell (NPC) graft. These grafts improve tissue reconstitution within the lesion site and are able to replace lost adult neural cell tissue with cells homologous to those in the pre-injured state. When a severed axon regrows into an NPC graft, it forms synapses on these developing progenitor cells. These NPCs then project axons that synapse onto other NPCs, eventually leading to circuit extension distal to the lesion site and improved functional recovery when compared to other treatments, although recovery of axon density caudal to the lesion site is still sparse (Kadoya et al., 2016; Fischer et al., 2020). Unfortunately, even with this new discovery that NPCs act as better graft that no longer requires experimental cAMP application (Kadoya et al., 2016.), full functional recovery is still unattainable following spinal cord injury (Fischer et al., 2020). In addition to the decreased axon density caudal to the graft, complete functional recovery is complicated by the “bridging” required for this regrowth. This “bridging” process takes a circuit that was once monosynaptic and creates a polysynaptic circuit reducing the speed and specificity at which signals from the motor cortex can reach their targets. In support of this bridging strategy, another group found that treatment with CLP290 to hyperpolarize lumbar inhibitory interneurons significantly improved hindlimb weight bearing stepping capacity through the revival of spared polysynaptic circuits following a bilateral double hemisection spinal cord injury at the T7 and T10 vertebrae (Chen et al., 2018). While this treatment did not induce any axon regeneration it demonstrates the potential for functional recovery through the creation of alternative circuits. There are many additional tissue grafts and experimental cell therapies for improving axon regeneration following SCI, for a comprehensive list (see Huang et al., 2021).

All neurons examined that project axons into the spinal cord have been shown to possess some regenerative potential in adult murine models. Yet, to date, no therapeutic approach has been able to produce full functional restoration of the deficits associated with CNS injury. The local milieu of the CNS is credited as a major factor dictating the dramatic shortcomings of CNS regeneration compared to that seen within the PNS, and yet, even in the context of modern treatment advancements, we still see differences between neuronal subtypes in both the rate and extent of regrowth. Is this extrinsic obstacle as critical as previously believed given the disparity in regeneration potential displayed across various CNS neuronal subtypes through an inhibitory environment that is consistent?

## Mammalian Neurons Revert to a Developing Phenotype Following Injury

Transcriptomic profiling using RNA sequencing techniques offers a new avenue to elucidate many of the genetic mechanisms underlying neuronal subtype-specific axon regeneration. By examining the transcriptome of individual neurons before injury and then at multiple timepoints following injury, we should be able to understand the gene expression events underlying axon regeneration. Furthermore, by comparing the results from different neuronal subpopulations, we should gain a greater understanding of why these populations display different propensities for regeneration.

An early study examining the transcriptome of regenerating DRG neurons in comparison to non-regenerating CNS neurons showed differences in the expression of various genes known to be associated with axon regeneration (Lerch et al., 2012; Dulin et al., 2015). This discovery provides potential insight to understanding the differences in regenerative potential shown by various neuronal subpopulations. Further studies used RNA-seq to examine specific pathways in detail. For example, Ma and colleagues found that CREB activated genes work in concert with AP-1 controlled genes to promote axon regeneration of cortical CNS neurons *in vitro* (Ma et al., 2014; Dulin et al., 2015). Studies such as these seek to understand axon regeneration at a pathway specific level in the hopes of identifying molecular targets for therapies.

Poplawski and colleagues recently published a study in which they used single-cell RNA-sequencing to examine the expression profile of individual CST neurons following spinal cord injury in mice at multiple timepoints throughout the regeneration process. They compared these profiles from mice that received NPC graft treatment to mice that had received only sham graft surgery after spinal cord injury. Surprisingly, both the NPC graft-treated animals and the lesion-only animals displayed the same initial transcriptional changes following injury, termed the “regeneration transcriptome.” The major difference seen between each group was the duration that the cell was able to maintain the regeneration transcriptome following injury. While some of the pathways activated were expected, such as those involved in axon guidance, others were surprising. Gene ontology analysis of the regeneration transcriptome revealed activation of proliferation, differentiation, and cell-cycle progression-related functions. These regenerating neurons had a transcriptome that was most highly similar to embryonic day 18 CST neurons, appearing that the injury was inducing “terminally differentiated” neurons to revert to an embryonic state (Poplawski et al., 2020).

Another key study using single-cell RNA-sequencing examined DRG neuron axon regeneration following a crush injury in the PNS (Renthal et al., 2020). Following injury application, the authors monitored the expression profiles of individual DRG neuronal subtypes to determine if they differ in their injury response. They were initially unable to identify the neuronal subpopulations after injury since the cells were downregulating genes classically used to distinguish those subtypes. While CST neurons reverted to a cell state closely resembling embryonic CST neurons, DRG neurons did not revert to this extent. Rather, DRG neurons increased the expression of transcription factors expressed during development which promote pluripotency. This increase was accompanied by the loss of cell identity indicative of reversion to a less differentiated state. Here in the periphery, we still see transcriptional reprogramming to a cell state resembling developmental neurons; however, the overlap between gene expression programs active during a specific developmental timepoint and those active during regeneration did not achieve statistical significance. Glial cells within the DRG did not experience a similar loss of identity, demonstrating that this is not a general cellular response to injury and is neuron-specific (Renthal et al., 2020). The findings from these two studies have the potential to alter the field’s approach to investigating axon regeneration, which has often focused on addressing individual barriers to regeneration. Rather than attempting to address specific extrinsic or intrinsic barriers, efforts can be redirected to initiating and prolonging neuronal reversion to a developmental state that would presumably lack intrinsic barriers.

Many studies in the past focused on identifying genes that are known to play a role in neuronal development and axon elongation with the hope of using those programs to promote regeneration. Such attempts have yielded only limited success over the course of decades. The dominant view has been that CNS neurons in adult mammals terminally differentiate following development and are incapable of fundamentally altering their transcriptional identity. With terminal differentiation in mind, researchers have been attempting to promote regeneration by blocking inhibition or activating regeneration-associated signaling pathways in the context of an adult neuron. The new findings by Poplawski et al. (2020) and Renthal et al. (2020) are some of the first to contradict this framework. It is important to note that while these redeveloping neurons may have overcome the intrinsic barriers to regeneration discussed above, they still face the same adult extracellular environment complete with an array of molecules inhibitory to axon regeneration. Still, results from these two studies could shift a major aim within the field from identifying specific RAGs that promote a slight increase in neurite outgrowth to identifying transcription factor hubs that promote or sustain neuronal reversion to an embryonic-like cell state.

If we were to assume that all neurons attempt a similar reversion following injury, we would be left with many of the same questions previously held in the field as well as some new ones. We still observe differences in the capacity of neuronal subpopulations to regenerate following injury, why is this? Perhaps certain neurons are able revert more quickly in response to injury or perhaps they are able to maintain this reverted state for longer. How do regeneration inhibitors affect this reverted state, are they promoting redifferentiation in some way? If all neurons undergo a process of redevelopment in order to respond to damage and facilitate recovery, it seems reasonable to assume that the local milieu of the PNS provides a better environment for neurons to maintain this reverted state. Does this warrant reexamination of factors previously identified to promote axon regeneration within this new context? Fitting these unanswered questions into a context of redevelopment may be the key to solving the puzzle of axon regeneration in the mammalian CNS.

## Some Central Nervous System Neuromodulatory Neurons Can Regenerate Their Axons Unaided Following Brain Injury in Adult Mammals

There is increasing evidence for spontaneous serotonergic neuron axon regrowth within the CNS following chemical or physical insults. Initial fixed tissue studies in the mammalian forebrain showed robust recovery of serotonin axon density following chemical lesioning with amphetamines as assessed using antibody staining against serotonin (O’Hearn et al., 1988; Molliver et al., 1990; Wilson and Molliver, 1994; Mamounas et al., 2000). A later investigation also used antibody staining against serotonin to demonstrate regrowth of serotonin axons in otherwise non-permissive environments including the subventricular zone and areas adjacent to a glial scar following a thermal injury—a feat not replicated by callosal fibers within the same cortical region (Hawthorne et al., 2011). Other studies examining recovery from spinal cord injury in rodents found that surviving serotonin axons exhibit significant compensatory sprouting rostral to the site of injury (Inman and Steward, 2003; Camand et al., 2004; Holmes et al., 2005; Hayashi et al., 2010). However, these experiments were unable to determine whether the recovery seen was truly due to regeneration. First, while the loss and subsequent recovery of serotonin immunoreactivity could indicate the retraction and regrowth of serotonin axons, this same effect could be observed if amphetamine treatment merely induced a long pause on the production of serotonin, effectively emptying the axons of serotonin for a period of time. Furthermore, because fixed tissue only displays a single moment in time, the recovery seen in these experiments may not be a result of bona fide regeneration, defined as growth originating from damaged axons, but rather the collateral sprouting of surviving axons (see Figure 1).

To directly address the issues inherent in examining axon regeneration using serotonin immunoreactivity as a measure, Linden and colleagues used long-term *in vivo* imaging in transgenic mice expressing eGFP selectively in serotonin neurons (Jin et al., 2016). Directly expressing eGFP within neurons, rather than staining the tissue with antibodies raised against serotonin, removes the concern that these axons may be emptying and refilling with serotonin following injury rather than retracting and regenerating. Furthermore, by examining regeneration *in vivo* using two-photon microscopy, directly monitoring the origin of axon recovery following parachloroamphetamine (PCA) treatment in individual damaged serotonin axons is possible. PCA treatment leads to the degeneration of serotonin axons back to the first presynaptic active zone (Molliver et al., 1990; Fuller, 1992). One week following PCA treatment there was a significant loss of serotonin axons within the neocortex; however, substantial recovery of serotonin axons was observed at both 3 and 6 months following PCA treatment. The rate of collateral axon sprouting in injured animals was the same as in controls, indicating that regeneration is indeed responsible for the recovery of axon density (Jin et al., 2016). The extent to which serotonin axons regenerated following PCA-induced serotonin degeneration is unprecedented compared to other studies examining recovery following CNS injury in wild-type animals. However, direct comparisons between recovery following a physical injury to that observed following a chemically induced serotonin axon-selective injury may not be appropriate. Physical injury not only affects all cells within the area, but also causes secondary injury cascades and glial scarring not seen following PCA treatment.

Though chemically induced neurodegeneration can occur in patients with long term amphetamine abuse (Halpin et al., 2014), this is not the most clinically relevant form of CNS injury. To address a more typical form of injury, Jin et al. (2016) further investigated this regrowth phenomenon of serotonin axons in physical injury models using transgenic mice expressing eGFP selectively within serotonin neurons. These mice received a stab wound to the somatosensory cortex in which a scalpel, oriented in the coronal plane, was inserted to transect serotonin axons running along their anterior to posterior trajectory. Unlike the amphetamine-induced injury, which selectively targets serotonin neurons leading to widespread axon degeneration back to the first presynaptic active zone, a physical stab injury damages all cell types in the area while only causing the proximal portions of serotonin axons to retract for tens of microns. Over the course of 12 weeks following injury, serotonergic axons traversed the glial scar and recovered regional axon density identical to mice that received a sham surgery. Using longitudinal *in vivo* imaging following stab injuries, axon regrowth directly from the severed ends of cut-injured axons was observed, providing direct evidence for axon regeneration (Jin et al., 2016). The same transgenic mouse model was also used to examine regeneration of serotonin axons following a controlled cortical impact (CCI) to induce traumatic brain injury (Kajstura et al., 2018). These mice received standardized impacts to the surface of the cortex, resulting in visible damage to cortical layers 1 through 4. Due to the extensive nature of the injury, axon regeneration was measured in the fixed tissue both anterior and posterior to the site. A week after injury, serotonin axon density is decreased only posterior to the CCI injury. However, a month after injury, the density of serotonin axons posterior to the injury is largely recovered despite the large crater created by the impact injury, which remains devoid of axons. This experiment indicates robust axon regeneration by serotoninergic neurons in the context of a more extensive injury is indeed possible (Kajstura et al., 2018).

When contemplating why serotonin axons seem to harbor an unusual ability to regrow without aid, we must consider how serotonin neurons are unique in their axonal structure, extensive neocortical innervation, and downstream neurotransmission. Serotonergic axons in the neocortex originate from cell bodies located in the rostral group of brainstem raphe nuclei. Their trajectory follows a C-shaped pattern from the brain stem along the base of the hypothalamus, through the medial forebrain bundle, up and around the anterior pole of the frontal lobe, and then anterior to posterior through cortical layers one and six as well as the underlying white matter; this trajectory defines these axons as some of the longest in the brain. Serotonergic axons running through these neocortical layers then branch off and dive to inferior locations, or climb to superior locations, to innervate all cortical layers (Jacobs and Azmitia, 1992). The inherent ability of serotonergic neurons to support these extremely long axons may provide them with the mechanistic framework to rebuild and repair them following axonal damage. However, simple mechanics might not entirely explain this phenomenon, since shorter projecting serotonergic axons are also capable of regrowth while other very long axons, such as CST axons, are unable to regrow following injury.

Another distinguishing quality of serotonergic neurons is that they mainly employ volume transmission, rather than conventional point-to-point synaptic transmission, as their primary method of signaling. In synaptic transmission, neurotransmitters such as glutamate, GABA, or glycine are released and activate receptors at classical synapses in which the presynaptic active zone faces a receptor-laden postsynaptic density lying across the narrow synaptic cleft. This form of point-to-point neurotransmission is built to convey information quickly (on a time scale of milliseconds to tens of milliseconds) and in a spatially restricted manner, with transmitter spillover usually restricted to only a few adjacent synapses. In volumetric transmission, there is no postsynaptic density bearing a high concentration of receptors facing the presynaptic active zone. Therefore, released serotonin diffuses in the extracellular space for a much greater distance in order to encounter dispersed receptors present on many cells within a larger volume (Séguéla et al., 1989; Bunin and Wightman, 1998, 1999; Fuxe et al., 2007; Kaushalya et al., 2008; Colgan et al., 2012). Due to the precision required for synapse-based signaling, if a damaged glutamate axon regrows, it must reinnervate its postsynaptic targets with great accuracy to reconstitute pre-injury function. By contrast, volume transmitting axons that regrow need only target the general area that was denervated, since their signaling is not so spatially or temporally constrained. Perhaps volume transmitting axons, like those that release serotonin, have the capacity to regrow after injury within the adult brain because such regrowth restores pre-lesion function without having to reform each of the precise connections of the previously injured axon.

The characteristics that may afford serotonin fibers the capability to regenerate unaided following injury—very long-distance axon projections and the use of volume transmission—are also shared by other neuromodulatory neurons, specifically norepinephrine- (also referred to as noradrenaline) expressing neurons. Like serotonin axons in the neocortex, norepinephrine axons also originate from cell bodies located in the brainstem in a region called the locus coeruleus, and they follow a similar C-shaped trajectory through the brain while also sending projections into the cerebellum and hindbrain (Beaudet and Descarries, 1978; Papadopoulos and Parnavelas, 1991; Berridge and Waterhouse, 2003; Sara, 2009; Shnitko and Robinson, 2014). Additionally, they also employ volume transmission to modulate target cells (Séguéla et al., 1990; Umbriaco et al., 1995; Fuxe et al., 2007). Previous studies show that lesions to the superior peduncle of the rat cerebellar cortex results in increased arborization of terminal norepinephrine fibers visualized *via* formaldehyde-induced fluorescence of catecholamines (Pickel et al., 1973). Chemical lesioning with the neurotoxin 6-hydroxydopamine (6-OHDA) led to a cortical decrease of norepinephrine, measured using a tritiated norepinephrine reuptake assay, in rats; these levels returned to normal after 3 months, suggesting regeneration or compensatory sprouting (Levin, 1983). Additionally, analysis of fixed tissue following 6-OHDA lesioning of the adult cat occipital cortex revealed degeneration and successive recovery of norepinephrine fibers over the course of 52 weeks, measured *via* antibodies against dopamine beta hydroxylase, the last enzyme in the norepinephrine metabolic pathway (Nakai et al., 1994). Following chemical damage with a selective norepinephrine neurotoxin (N-(2-chloroethyl)-N-ethyl-2-bromobenzylamine, known as DSP-4), norepinephrine immunoreactivity is first lost and then subsequently recovered by 12 months post-damage (Fritschy and Grzanna, 1992).

A recent study showed reduction of norepinephrine axon density following CCI or the application of a cortical stab wound at 1 week and 1 month following injury. Much like serotonin axons, axon density slowly returned over the course of several months to levels indistinguishable from the cortical innervation observed in control mice (Dougherty et al., 2020). As with the initial evidence of serotonergic regeneration, these fixed tissue studies are unable to provide definitive evidence of regeneration vs. collateral sprouting, and so long-term *in vivo* imaging is still required.

Together these results suggest that both serotonin and norepinephrine neurons in the CNS possess the intrinsic ability to regrow their axons in the adult brain. It is not known whether this phenomenon is shared by any other class of neuron, or if this ability is a result of their unique characteristics. Further studies need to be conducted to determine if other neuromodulatory axons can regrow after injury, or if CNS regeneration is exclusive to serotonin and norepinephrine-expressing neurons.

## Conclusion: Implications of Redevelopment and Regeneration of Neuromodulatory Axons

The discovery that serotonin and norepinephrine neurons can regenerate axons projecting throughout the cerebrum raises several interesting questions. Over the past several decades, extensive work has shown that the PNS offers a much more effective environment for regeneration than the CNS. Indeed, limitations of PNS axon regeneration through a CNS graft and the effectiveness of promoting CNS axon regeneration through the application of a PNS graft clearly demonstrates this difference (Aguayo et al., 1978; Richardson et al., 1980; David and Aguayo, 1981; Richardson et al., 1984). Subsequent work showing the increased effectiveness of both bone marrow stromal cell and also NPC-based grafts improve upon the pro-growth effects observed following application of PNS grafts (Jin et al., 2002; Vavrek et al., 2007; Lu et al., 2012; Lee et al., 2013; Kadoya et al., 2016; Fischer et al., 2020). The recent findings that axon regeneration may be facilitated by the reversion to an embryonic-like cell state that can be maintained for longer periods by the addition of an NPC graft also fits within this narrative. These studies show that CNS neurons maintain an ability to regenerate following axonal injury given the right environment to promote an internal growth state. This permissive environment is present within axon tracts of the PNS but not the CNS.

The regenerative capabilities of serotonin axons from the dorsal/median raphe and norepinephrine axons from the locus coeruleus into the cerebral cortex show that adult mammalian CNS neurons are indeed able to regenerate axons following injury through an environment that has traditionally been considered non-permissive. Several groups have observed the ability of axons from serotonin and norepinephrine-expressing neurons to regenerate over long distances following traumatic brain injury, a cortical stab wound, or selective chemical lesions (O’Hearn et al., 1988; Molliver et al., 1990; Fritschy and Grzanna, 1992; Nakai et al., 1994; Wilson and Molliver, 1994; Mamounas et al., 2000; Inman and Steward, 2003; Camand et al., 2004; Holmes et al., 2005; Hayashi et al., 2010; Jin et al., 2016; Kajstura et al., 2018; Dougherty et al., 2020). The magnitude of regeneration observed within these studies is beyond that of any other CNS axon regeneration observed to date and requires no experimental intervention. These regenerated serotonergic and noradrenergic axons are present in densities equivalent to what is observed in uninjured tissue. Further, these regenerated axons are capable of neurotransmitter release by 6 months following injury, as detected by fast-scan cyclic voltammetry, albeit evoked by pulse trains far beyond those which occur physiologically. Remarkably, this regeneration results in mice that are indistinguishable from their littermate controls in several behavioral tasks (Jin et al., 2016).

Multiple approaches have been shown to improve behavioral recovery following spinal cord injury, neural progenitor cell grafting being the most successful; however, full recovery including the more complicated movements has yet to be observed. It should be noted that the role these modulatory neurotransmitters play in behavior is much more subtle than the role that CST neurons play in movement. Nevertheless, regeneration on this scale has yet to be seen under any circumstance in the spinal cord or the optic nerve.

Serotonin and norepinephrine axon regeneration through the cerebral cortex shows several distinct features from axon regeneration induced in the spinal cord, optic nerve, or that seen in the PNS. First, cortical serotonergic and noradrenergic axons are able to regenerate through the glial scar created by a cortical stab wound (Jin et al., 2016; Dougherty et al., 2020). Following spinal cord injury, no axon is able to pass through the glial scar unaided. Furthermore, when glial scar tissue is grafted into a PNS lesion, DRG axons are unable able to regrow through the graft (Davies et al., 1997; Davies et al., 1999). Interestingly, serotonin and norepinephrine axons are unable to pass through the glial scar of a spinal cord transection unaided but are able to do so in the cerebral cortex (Lee et al., 2010). Somehow, these neuromodulatory axons within the brain are able to ignore the inhibitory cues inherent to the CNS. Are there intrinsic differences between the subpopulations of neuromodulatory neurons that project into the spinal cord and those that project cranially? For example, the cranial projecting neurons may display variation in key signal transduction pathways that inhibit regeneration which eliminates the potential for that signaling. It is also possible that the environment within the adult mammalian brain is more permissive for axon regeneration than previously believed, at least for neuromodulatory neurons.

Another difference between DRG and serotonergic axon regeneration is that serotonergic axons do not regrow along the exact path from which they retracted, as is observed for regenerating PNS axons, indicating that these neurons use different axon guidance mechanisms. Finally, following spinal cord or PNS injury, there are many more branches that extend from damaged axons than the final number that will eventually innervate their targets. Axon branches that do not receive growth factor signaling from that target organ, or from a graft following spinal cord injury, are eliminated. This resembles the overgrowth and pruning process seen in the developing nervous system. Monitoring regrowth using *in vivo* 2-photon imaging of serotonergic axons during regeneration through the cerebral cortex did not reveal similar overgrowth and pruning.

Given the observed differences between serotonergic axon regeneration and DRG axon regeneration, it is possible that serotonergic and norepinephrine neurons regrow using different regeneration programs than PNS neurons. The experiments conducted by Poplawski et al. (2020) and Renthal et al. (2020) indicate that CST neurons following spinal cord injury and implementation of an NPC graft, as well as DRG neurons following peripheral crush injury, undergo reversion to a developmental-like phenotype to promote recovery and axon regeneration. This hypothesis is further supported by the overgrowth and pruning of axonal branches following these injuries as well as the success many labs have found in promoting regeneration through mTOR, a pathway highly active during development (Nieuwenhuis and Eva, 2022). Developing cells are much more resilient to damage than adult cells and thus this reversion to a developmental phenotype may be part of the common neuronal injury response.

One characteristic of developing neurons is increased neurite sprouting, which would explain the arborization and pruning observed during PNS regeneration. The increased expression of growth factors such as BDNF seen in the periphery likely provides signaling necessary for DRG neurons to maintain a reverted developmental phenotype long enough to reestablish synaptic connection to the target organs. Graft implementation following a spinal cord injury approximates this same process, adding developmental tissue to the injured neurons providing the molecular signaling sufficient to maintain this reverted state for longer. The observed differences described above indicate that serotonergic neurons may not undergo this same process of “redevelopment,” raising the possibility that they may be using regenerative programs distinct from that of the PNS. If this is the case, new avenues of research for the field in CNS axon regeneration present the opportunities to identify and exploit these novel regenerative programs. Perhaps attempting to recapitulate the PNS is not the only, or best, approach to achieve clinically relevant regeneration within the CNS.

Single cell expression profiling experiments should begin to answer some of these questions. Okaty et al. (2020) recently published an in-depth single cell transcriptomic atlas of serotonin neurons within the dorsal raphe of young adult mice aged 6–10 weeks. They identified up to 14 distinct neuronal subtypes within the region, all expressing serotonin. Broadly, these subtypes can be split into two groups, those that co-express GABA and those that co-express glutamate (Okaty et al., 2020). This discovery calls into question the regeneration of “serotonin” neurons as a broad definition. Do all of these subtypes possess the same capacity for regeneration or are only a few competent to regenerate? If these different subtypes of serotonin neurons are all competent to regenerate, will they regress to a developing phenotype? If so, to what extent will this regression occur, will they lose their individual identities as seen within different subtypes of DRG neurons? Expression profiling before and at multiple time points after axon injury will reveal the “regeneration transcriptome” of serotonin and norepinephrine neurons which can be compared to the transcriptomes of developing serotonin (Wyler et al., 2016) and norepinephrine neurons.

Comparing the transcriptome of regenerating neuromodulatory neurons to those of DRG and CST neurons at equivalent timepoints will determine whether serotonin and norepinephrine neurons undergo a similar reversion process in order to facilitate recovery. Results from these experiments could change our view of how to search treatments directed toward traumatic brain and spinal cord injuries. Rather than attempting to induce expression of the ideal transcriptional programs in CNS associated with peripheral nerve regeneration, efforts designed to maintain a “redevelopment” phenotype specific to CNS neurons, or perhaps a program unique to nerumodulatory neurons will be most productive. How does the unprecedented regenerative capacity of neuromodulatory axons in the brain fit into the overall narrative of redevelopment? If neuromodulatory neurons facilitate regeneration through redevelopment, then extending this period of redevelopment should be a central focus of research in the search for therapies. If, however, neuromodulatory neurons use alternative mechanisms, then these hold the promise for recapitulating regenerative programs proven to work within the CNS. It is important to note that neuromodulatory neurons are quite different from other neurons in both structure and function and thus a deep understanding of their regenerative states is required in order to determine whether their regenerative programs may be utilized in other systems.

## Author Contributions

PC conceived the review and wrote sections “Axons in the Adult Mammalian Central Nervous System Do Not Regenerate Following Injury,” “Graft Transplants Promote Axon Regeneration Following Spinal Cord Injury”, “Mammalian Neurons Revert to a Developing Phenotype Following Injury”, and “Conclusion: Implications of Redevelopment and Regeneration of Neuromodulatory Axons” and conceptualized the figures. HJ wrote sections “Axons in the Adult Mammalian Central Nervous System Do Not Regenerate Following Injury” and “Some Central Nervous System Neuromodulatory Neurons Can Regenerate Their Axons Unaided Following Brain Injury in Adult Mammals”. SD wrote section “Some Central Nervous System Neuromodulatory Neurons Can Regenerate Their Axons Unaided Following Brain Injury in Adult Mammals”. HJ created the figures. PC, HJ, and SD edited the manuscript. All authors contributed to the article and approved the submitted version.

## Conflict of Interest

The authors declare that the research was conducted in the absence of any commercial or financial relationships that could be construed as a potential conflict of interest.

## Publisher’s Note

All claims expressed in this article are solely those of the authors and do not necessarily represent those of their affiliated organizations, or those of the publisher, the editors and the reviewers. Any product that may be evaluated in this article, or claim that may be made by its manufacturer, is not guaranteed or endorsed by the publisher.
